# Tooth Reattachment and Palatal Veneer on a Multidisciplinary Approach of Crown Fractures in Upper Central Incisors

**DOI:** 10.1155/2017/4702635

**Published:** 2017-09-26

**Authors:** Vanessa Machado, Ricardo Alves, Luísa Lopes, João Botelho, José João Mendes

**Affiliations:** ^1^Department of Oral Rehabilitation, Instituto Superior de Ciências da Saúde Egas Moniz, Monte da Caparica, Almada, Portugal; ^2^Department of Periodontology, Instituto Superior de Ciências da Saúde Egas Moniz, Monte da Caparica, Almada, Portugal; ^3^Department of Pediatrics, Instituto Superior de Ciências da Saúde Egas Moniz, Monte da Caparica, Almada, Portugal; ^4^Department of Dentistry, Instituto Superior de Ciências da Saúde Egas Moniz, Monte da Caparica, Almada, Portugal

## Abstract

Dental trauma is more common in young patients and its sequelae may have great impact on the esthetics, functions, and phonetics. This paper reports a case of trauma in both central incisors in a young 17-year-old patient who was treated using adhesive tooth fragment reattachment on tooth 2.1 and a palatal indirect composite veneer on tooth 1.1. Regarding the available literature and fracture extension, the treatment approach proposed for this case provided good functional and esthetic outcomes. Clinical and radiographic results after 1 year were successful. This case demonstrates the importance of establishing a multidisciplinary approach for successful dental trauma management.

## 1. Introduction

Dentoalveolar trauma is very common in children and adolescents and can result from an accidental fall, a traffic accident, contact sports, or play. Dental trauma can cause fractures in the maxillary anterior teeth and these subsequently lead to esthetic, functional, and phonetic problems [1, 2].

Crown fractures account for the majority of dental trauma in the permanent dentition (26–76% of dental injuries) [3], while crown-root fractures represent only 0.3–5% [4] and require a complex and multidisciplinary treatment [5].

This clinical report describes an unusual case of fragment reattachment in a crown-root fracture with involvement of the biologic width on the upper left central incisor and the restoration of an uncomplicated crown fracture with a composite palatal veneer of the upper right central incisor, with one-year successful follow-up.

## 2. Case Report

**Figure 1:**
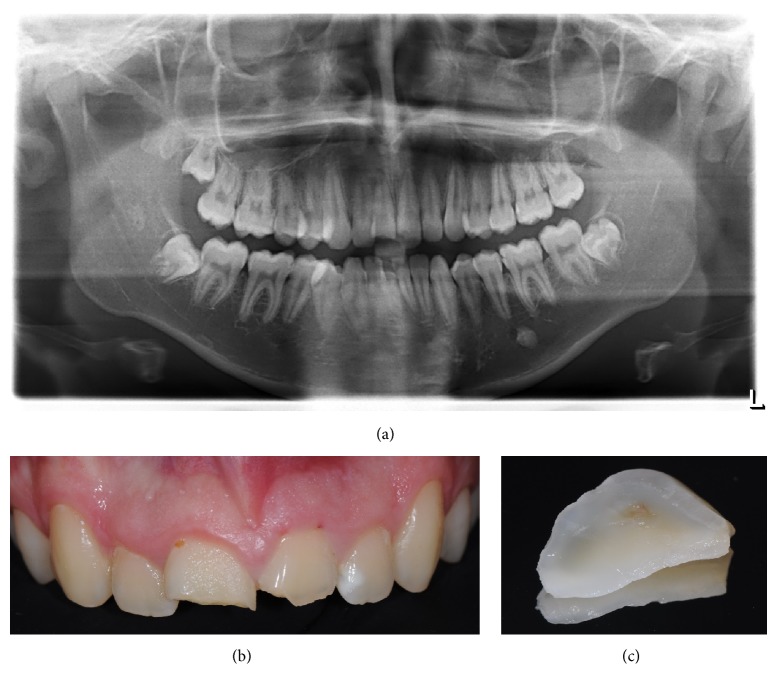
(a) Initial orthopantomography. (b) Preoperative clinical view. (c) Tooth fragment.

A 17-year-old male patient presented to Egas Moniz University Clinic (Egas Moniz, Health Sciences Institute) for an urgent consult thirty days after craniofacial trauma (Figure 1(b)). The patient had no significant medical history. The upper left central incisor (2.1) had a complicated crown-root fracture with palatal involvement of the biologic width and the fragment (Figure 1(c)) was attached to the junctional epithelium and connective tissue. The upper right central incisor (1.1) had a fracture line located in the middle third of the tooth and the fragment was lost. Tooth 2.1 showed pulp involvement, with sensitivity tests (thermal and electrical pulp tests) indicating pulp necrosis, and the tooth showed grade I mobility. On the contrary, tooth 1.1 showed no evidence of pulpal exposure or periodontal injury. There were no signs of soft tissue laceration or alveolar bone fracture evidence. The radiographic examination revealed full root development and absence of root fracture of both teeth (Figure 1(a)).

## 3. Rehabilitation of Tooth 2.1

**Figure 2:**
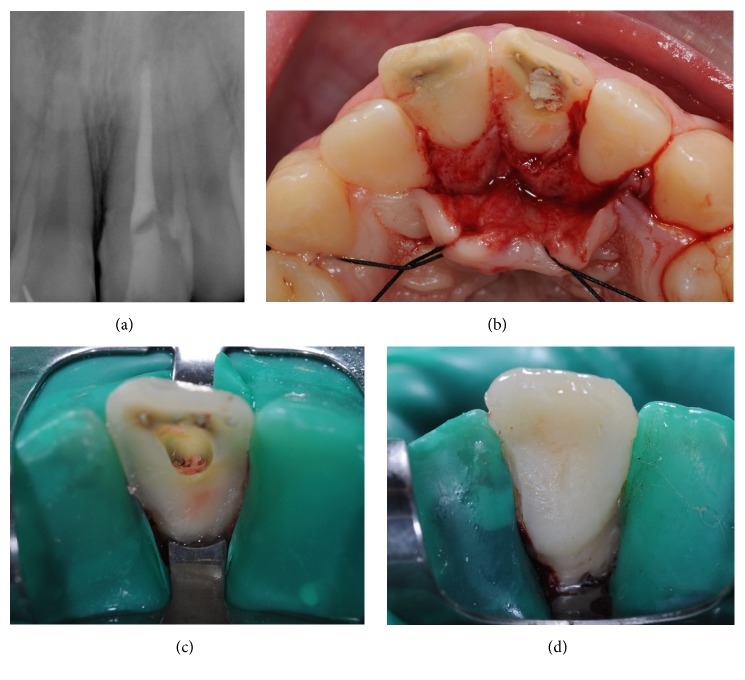
(a) Final periapical radiograph after pulpectomy. (b) Clinical aspect during the exploratory surgery showing the extension of the complicated crow-root fracture, invading the biological width in the palatal aspect. (c) After gingival flap and osteotomy, a rubber dam was placed. (d) Final aspect after fragment reattachment and polishing.

After obtaining the patient's consent, we anesthetized the patient locally (2% lidocaine with 1 : 80000 adrenaline), removed the fragment, and performed pulpectomy of tooth 2.1. The tooth fragment was placed in a saline solution (NaCl) until the adhesive procedure. Thereafter, we performed a pulpectomy in a single-session endodontic treatment. We used hand K files (MANI Inc., Tochigi, Japan) (Table 1) up to the apical size 45 followed by step-back instrumentation. Before final closure of the canal, the canals were irrigated with 5.25% sodium hypochlorite and 17% liquid EDTA; both were activated with manual dynamic irrigation. The canals were then dried with sterile paper points. Obturation was done with gutta-percha (Dentsply Maillefer, Ballaigues, Switzerland) and root canal sealer (AH Plus, Dentsply, Konstanz, Germany) using lateral condensation technique (Figure 2(a)).

It was necessary to perform an intrasulcular incision followed by a gingival flap (Figure 2(b)) to allow rubber dam isolation. When exposing the fracture margin, it was observed that the fracture line was located intraosseously, invading the biologic width. Therefore, osteotomy and osteoplasty were necessary in the palatal region, removing approximately 1 mm of bone tissue. Rubber dam isolation was performed using a #212 retraction clamp (Hu-Friedy, Chicago, USA) in order to expose the fracture margin and to keep the area clean and dry, providing favorable conditions for the restorative treatment (Figure 2(c)).

**Figure 3:**
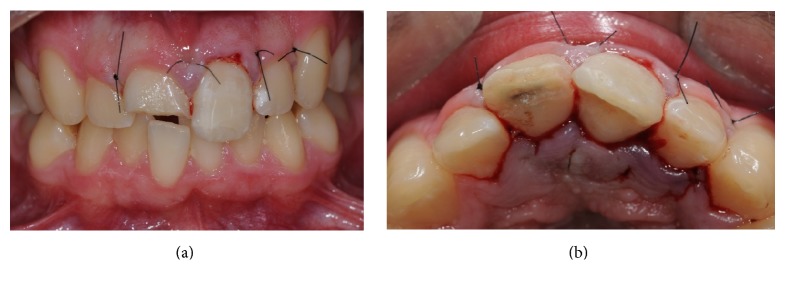
(a) Buccal aspect of the sutures. (b) Palatal aspect of the sutures.

After repositioning the tooth fragment, it was possible to observe excellent margin adaptation. Before reattaching the fragment, gutta-percha was covered with resin-modified glass ionomer (Vitrebond, 3M ESPE, USA) (Figure 2(d)). Therefore, we reattached the fragment using an adhesive procedure, described in Table 2. After removing the glycerine gel, we finished with diamond burs and polishing discs (Sof-Lex, 3M, USA) and checked for occlusal adjustments. After rubber dam removal, the gingival flap was repositioned and the papillae were sutured with 4.0 silk braided nonabsorbable suture material (SMI, Belgium) (Figures 3(a) and 3(b)).

## 4. Rehabilitation of Tooth 1.1

**Figure 4:**
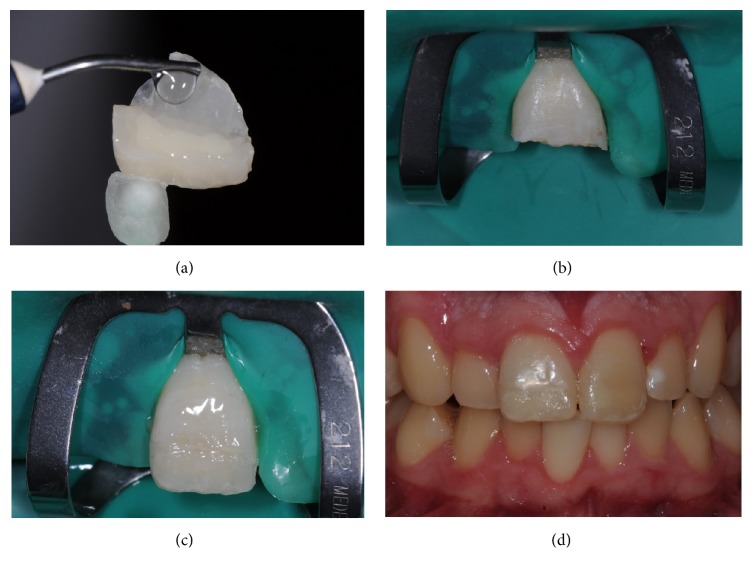
(a) Veneer surface conditioning. (b) Preparation for veneer adhesive procedure on tooth 1.1. (c) After adhesion palatal veneer in 1.1. (d) Clinical aspect of buccal view after rehabilitation of 2.1 and 1.1.

At the second appointment, due to the size of the lost fragment, we planned a palatal indirect composite veneer. We took impressions (Alginate; 3M ESPE, United States) of both arches. Type IV die stone (Elite Rock; Zhermack, Badia Polesine, Rovigo, Italy) was poured, and the casts were mounted in a semiadjustable articulator. We made the palatal veneer/onlay using a hybrid composite (Filtek Supreme XTE, Auckland, New Zealand) (Figure 4(a)).

At the third appointment, the veneer was then intraorally evaluated to assess marginal fit and esthetics before the adhesive procedure. With rubber dam placed (Figure 4(b)), we performed the palatal veneer adhesion, described in Table 2 (Figure 4(c)). After removing the glycerine gel, we finished with diamond burs and polishing discs (Sof-Lex, 3M, USA) and checked for occlusal adjustments (Figure 4(d)).

**Figure 5:**
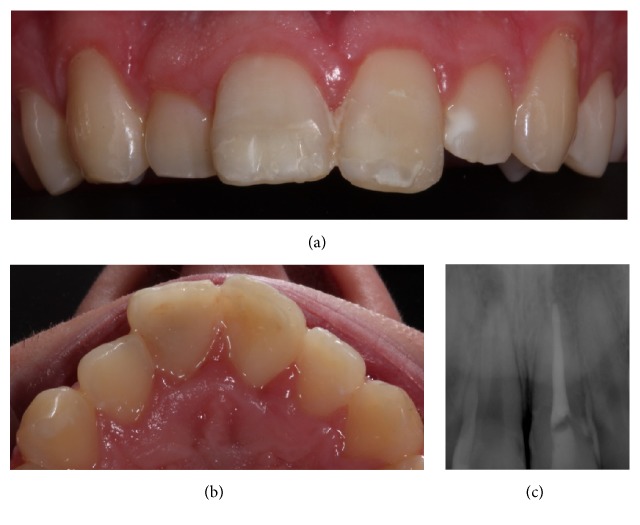
(a) Clinical aspect of buccal view after 1-year follow-up. (b) Clinical aspect of palatal view after 1-year follow-up. (c) The periapical radiograph after 1-year follow-up.

After 1-year follow-up, thermal and electrical pulp tests [9] showed pulp vitality of tooth 1.1. Furthermore, clinical (Figures 5(a) and 5(b)) and radiographic (Figure 5(c)) findings of both teeth presented restorations with good adaptation and no color change, absence of radiographic signs of root resorption, no mobility, no pocket formation or gingival recession and absence of painful symptomatology, and tenderness to percussion.

## 5. Discussion

Various treatment approaches have been proposed for patients with tooth fractures. For years, crowns have been the treatment of choice to restore anterior teeth after a trauma event, but this option is highly invasive and requires an extensive tooth preparation, which may have possible adverse effects [10].

Treatment approaches can be changed depending on the level of fracture line and the amount of remaining root. The options for such fractures include reattachment, fragment removal, and immediate restoration; restoration after gingivectomy or osteotomy; forced orthodontic extrusion; forced surgical extrusion; vital tooth submergence; resin or ceramic crowns; and direct or indirect resin composite restoration [1, 11–14]. Several factors influence the management of tooth fractures, including the extent and pattern of fracture, restorability of the fractured tooth, secondary injuries, the presence or absence of the fractured tooth fragment and its condition (the fit between the fragment and the remaining tooth structure), occlusion, esthetics, cost, and prognosis [15].

Furthermore, complicated crown fracture is a fracture involving enamel, dentin, and pulp exposure and therefore requires the treatment of the pulp by pulp capping, pulpotomy, or pulpectomy. Technical, biological, and esthetical problems are exacerbated when the fracture extends subgingivally and impinges on the biologic width, as access to the most cervical margin of the fracture and adequate isolation of the operating field area are difficult to achieve [13]. If the fracture extends further subgingivally, flap surgery, combined with osteoplasty/osteotomy procedures, is typically required [1, 16, 17]. After flap surgery, teeth fragments can be reattached using adhesive systems [1, 18]. It is a safe and effective technique, although it may compromise bone support of the adjacent tooth. Besides that, if not performed carefully, it can reduce the cervical diameter of the tooth [5].

The remarkable evolution of adhesive systems and resin composites has made reattachment of tooth fragment a procedure that is no longer a provisional restoration but rather a restorative treatment, offering a favorable and durable prognosis. In a reattachment procedure, the use of an intermediate resin composite layer significantly increases the fracture strength recovery [19]. Therefore, we used heated Z100 because the elastic modulus (16 GPa) is similar to that of dentin (18.6 GPa) and presents excellent “bondability” properties [6, 20].

Generally, palatal aspect loss or fracture of the maxillary anterior teeth leads to a substantial loss and weakening of tooth structure.

As stated by Vailati and Belser [21], the mesial and distal marginal ridges of the anterior teeth give structural strength, thus representing a framework for enamel. Therefore, the removal of these marginal ridges, for example, in a crown preparation, could dramatically compromise the tooth flexibility even more in teeth with a past history of trauma. This way, to be minimally invasive, we opted for an indirect palatal veneer [21]. Fabricating the palatal onlay in the laboratory presents important advantages, including higher wear resistance and precision during the creation of the final form [22]. It is difficult to visualize the optimal final morphology of the teeth, particularly while restoring at this stage only the palatal side with rubber dam in place [21].

This case represents an ultraconservative approach in which we used adhesive procedures, reattaching the tooth fragment on tooth 2.1 and adhering a palatal veneer on tooth 1.1. In both, very good results were obtained after 1-year follow-up. Thus, both techniques are viable and restore function and esthetics with a very conservative approach and should be considered as an alternative treatment for patients with fractured anterior teeth.

## Figures and Tables

**Table 1:** Materials used.

#212 retractor clamp	Hu-Friedy (Chicago, USA)
CoJet	3M (Seefeld, Germany)
Phosphoric acid 37% (etching gel)	Dentaflux (Madrid, Spain)
OptiBond FL (adhesive)	Ker (Orange, California)
Filtek Z100™ (resin cement)	3M (USA)
Filtek™ Supreme XTE	3M ESPE (Auckland, New Zealand)
Glycerine gel	Liquid Strip, Ivoclar Vivadent
Sof-Lex™ (polishing discs)	3M ESPE (St. Paul, MN, USA)
Vitrebond	3M ESPE (USA)
AH Plus	Dentsply (Konstanz, Germany)
Gutta-percha	Dentsply Maillefer (Ballaigues, Switzerland)

**Table 2:** Conditioning protocol of the tooth and palatal indirect composite [5–7].

Sequence of conditioning in palatal indirect veneer	Sequence of conditioning in the tooth/fragment
Sandblasting with CoJet (5 s)	Acid etching enamel and dentin (15 s) (37% H_3_PO_4_)
Rinsing and drying (30 s)	Rinsing and drying (30 s)
Acid etching enamel and dentin (15 s) (37% H_3_PO_4_)	Adhesive resin (OptiBond FL) application
Rinsing and drying (30 s)	No photopolymerization
Ultrasonic vibration with distilled water (4 minutes)	Heated Z100 (3M, USA)
Silane application in palatal indirect composite	Removal of excess of Z100
Adhesive resin (OptiBond FL) application	Photopolymerization (40 s each side)
No photopolymerization	Glycerine gel application and photopolymerization at buccal, oral, and proximal sides (40 s each) [8]

## Additional Points

Fragment reattachment and palatal indirect veneer can be used to treat fractured teeth successfully following trauma in children and adolescents.
