# Corrigendum to “Tooth Reattachment and Palatal Veneer on a Multidisciplinary Approach of Crown Fractures in Upper Central Incisors”

**DOI:** 10.1155/2017/9067393

**Published:** 2017-12-03

**Authors:** Vanessa Machado, Ricardo Alves, Luísa Lopes, João Botelho, José João Mendes

**Affiliations:** ^1^Egas Moniz Cooperativa de Ensino Superior, Monte da Caparica, Almada, Portugal; ^2^Department of Periodontology, Instituto Superior de Ciências da Saúde Egas Moniz, Monte da Caparica, Almada, Portugal; ^3^Department of Pediatrics, Instituto Superior de Ciências da Saúde Egas Moniz, Monte da Caparica, Almada, Portugal; ^4^Department of Dentistry, Instituto Superior de Ciências da Saúde Egas Moniz, Monte da Caparica, Almada, Portugal

In the article titled “Tooth Reattachment and Palatal Veneer on a Multidisciplinary Approach of Crown Fractures in Upper Central Incisors” [1], there was a mistake in the first affiliation. The corrected affiliations are shown above.
